# Region-specific brain area reductions and increased cholecystokinin positive neurons in diabetic OLETF rats: implication for anxiety-like behavior

**DOI:** 10.1186/s12576-020-00771-0

**Published:** 2020-09-16

**Authors:** Ryosuke Ochi, Naoto Fujita, Natsuki Goto, Son Tien Nguyen, Duc Trung Le, Kojiro Matsushita, Taketoshi Ono, Hisao Nishijo, Susumu Urakawa

**Affiliations:** 1 Department of Musculoskeletal Functional Research and Regeneration, Graduate School of Biomedical and Health Sciences, Hiroshima University, 1-2-3 Kasumi, Minami-ku, Hiroshima, 734-8553 Japan; 2 Department of Rheumatology and Endocrinology, 103 Military Hospital, Vietnam Military Medical University, 160, Phung Hung Street, Phuc La, Ha Dong, Hanoi, 12108 Vietnam; 3 Department of Neurology, 103 Military Hospital, Vietnam Military Medical University, 160, Phung Hung Street, Phuc La, Ha Dong, Hanoi, 12108 Vietnam; 4 Department of Mechanical Engineering, Facility of Engineering, Gifu University, 1-1 Yanagido, Gifu, 501-1193 Japan; 5 System Emotional Science, Graduate School of Medicine and Pharmaceutical Sciences, University of Toyama, Sugitani 2630, Toyama, 930-0152 Japan

**Keywords:** Imbalance, Cholecystokinin, Parvalbumin, Brain area, Type 2 diabetes, Limbic system

## Abstract

Metabolic disorders can induce psychiatric comorbidities. Both brain and neuronal composition imbalances reportedly induce an anxiety-like phenotype. We hypothesized that alterations of localized brain areas and cholecystokinin (CCK) and parvalbumin (PV) expression could induce anxiety-like behavior in type 2 diabetic Otsuka Long-Evans Tokushima fatty (OLETF) rats. Twenty-week-old OLETF and non-diabetic Long-Evans Tokushima Otsuka (LETO) rats were used. The areas of corticolimbic regions were smaller in OLETF rats. The densities of CCK positive neurons in the lateral and basolateral amygdala, hippocampal cornu ammonis area 2, and prelimbic cortex were higher in OLETF rats. The densities of PV positive neurons were comparable between OLETF and LETO rats. Locomotion in the center zone in the open field test was lower in OLETF rats. These results suggest that imbalances of specific brain region areas and neuronal compositions in emotion-related areas increase the prevalence of anxiety-like behaviors in OLETF rats.

## Introduction

Metabolic disorders, such as obesity and type 2 diabetes, induce several neurological comorbidities not only in the peripheral nervous system, but in the central nervous system as well. Namely, psychiatric disorders such as cognitive impairment, depression, and anxiety are attracting considerable attention [1, 2]. Psychiatric disorders, cognitive impairment and anxiety, are found to be not only induced by type 2 diabetes, but by obesity and metabolic syndrome as well [3, 4]. Patients with type 2 diabetes and anxiety reportedly had an increased risk of mortality compared with non-diabetic and non-anxious individuals [5]; therefore, the coincidence of anxiety in patients with type 2 diabetes is a serious problem. Since obesity and metabolic syndrome often precede the onset of type 2 diabetes, the development of treatments and early prevention would benefit from understanding anxiety’s etiology in the obese patients as well as those at early stages of type 2 diabetes.

In the central nervous system, appropriate balances in certain aspects play critical roles regarding both physiological and psychiatric functions. Imbalances of excitation and inhibition in synaptic transmission, neural circuits, signaling pathways, and brain composition are implicated in anxiety-like phenotypes in autism spectrum disorder and schizophrenia [6–8]. Therefore, anxiety in type 2 diabetes may also be associated with imbalances in brain morphology and histology.

Volume reduction in some brain regions is one of the neuroanatomical mechanisms of anxiety in patients with type 2 diabetes. Previously, patients with type 2 diabetes with anxiety and depression exhibited decreased gray matter volume as compared to patients with diabetes without anxiety and depression [9]. In addition, patients with an anxiety disorder also showed reductions of both gray and white matter volumes in some emotion-related brain regions, which correlated with their anxiety severity [10]. These findings suggest that volume reductions in emotion-related brain regions induce anxiety in type 2 diabetes.

Consistent with the findings in humans, results from type 2 diabetes animal models have also shown increased anxiety-like behavior. Otsuka Long-Evans Tokushima fatty (OLETF) rats are a type 2 diabetic model established by selective breeding of spontaneous diabetic rats [11]. OLETF rats develop type 2 diabetes with age due to hyperphagia. We previously showed that almost all OLETF rats exhibited hyperglycemia and hyperinsulinemia by the age of 20 weeks. After the age of 30 weeks, OLETF rats demonstrated a mixture of hyperinsulinemic and hypoinsulinemic symptoms, showing that the pathologies of type 2 diabetes were still progressing after the age of 20 weeks in OLETF rats [12–14]. Between the age of 10 and 20 weeks, OLETF rats exhibited an increased anxiety-like behavior, as demonstrated by decreased entries and time spent in the open arms of the elevated plus maze test, decreased time spent in the light box of the light-dark box test, and increased latency to leave the center area and time spent in the shadow area in the open field test [15–17]. Therefore, OLETF rats at the age of 20 weeks should be appropriate as a model for early-stage type 2 diabetes with anxiety.

It has been suggested that increased anxiety-like behavior in OLETF rats arises from type 2 diabetes and/or the deficit of cholecystokinin (CCK)-1 receptor [15–17]. CCK is one of the neuropeptides expressed in the central nervous system [18]. Two subtypes of CCK receptors have been identified: CCK-1 and CCK-2; however, the CCK-1 receptor gene knockout mice did not modify anxiety-like behavior [19], whereas the injection of a selective CCK-2 receptor antagonist into the cerebral ventricles and hippocampus decreased anxiety-like behavior [20, 21]. Therefore, increased anxiety-like behavior in OLETF rats could be induced not only by the deficit of CCK-1 receptor, but also from altered expression of CCK and/or the CCK-2 receptor. Although the expression of the CCK and CCK-2 receptor mRNA in the cerebral cortex has been reported to be comparable to that in control Long-Evans Tokushima Otsuka (LETO) rats [22], it remains to be determined whether the expression of CCK and the CCK-2 receptor are altered in the limbic system of OLETF rats.

CCK is expressed in pyramidal cells and basket cells [23–25]. Lassorn et al. reported that inhibitory neurons (glutamic acid decarboxylase 67 [GAD67] positive neurons) are reduced in the cortex and striatum in an animal model of type 2 diabetes [26]. However, little is known about whether type 2 diabetes affects the expression of CCK in inhibitory and excitatory neurons. Although both inhibitory and excitatory CCK positive neurons are associated with emotional behaviors such as anxiety and depression [25, 27], inhibitory CCK positive neurons might be specifically altered and associated with anxiety-like behavior in OLETF rats.

Basket cells are neurochemically divided into two types in the amygdala: CCK or parvalbumin (PV) expressing neurons [28, 29]. Both CCK and PV positive neurons in the amygdala have been classified as large or small types according to the size of somata [23, 30]. Although functional differences of CCK and PV positive neurons due to the morphological properties are not fully understood, some reports indicate the relationships between morphological and physiological properties: the morphological properties of CCK positive neurons in the amygdala are reportedly associated with neurochemical classification, large CCK positive neurons co-express calbindin and neurokinin 1 receptor, whereas small CCK positive neurons co-express calretinin and vasoactive intestinal polypeptide [23, 31]. We previously showed that small PV positive neurons, but not large PV positive neurons, in the amygdala are involved in anxiety-like behavior [30]. Therefore, large and small types of CCK and PV positive neurons in the amygdala might be differentially altered and associated with anxiety-like behavior in OLETF rats.

Collectively, these findings led us to hypothesize that OLETF rats may have imbalances in the brain areas and composition of CCK and PV expression in the limbic system related to increased anxiety-like behavior in the early stage of type 2 diabetes. To address this hypothesis, we investigated brain weight and areas, the densities of CCK and PV positive neurons in the limbic system, and anxiety-like behavior, and analyzed the relationships between these alterations.

## Methods

### Animals

Twenty-week-old male OLETF rats (*n* = 6) and age-matched control LETO rats (*n* = 5) were used. We housed three rats per cage that were maintained under controlled temperature conditions (22 ± 2 °C) and a 12-h light-dark cycle (lights on from 8:00 to 20:00). Food and water were provided ad libitum; moreover, the same rats were used in our recent publication [14]. This study was approved by the Institutional Animal Care and Use Committee of Hiroshima University (A16-5) and was performed according to the Hiroshima University Regulations for Animal Experimentation. All experiments were conducted in accordance with the National Institute of Health Guidelines for the Care and Use of Laboratory Animals.

### Oral glucose tolerance test

At the age of 20 weeks, an oral glucose tolerance test was performed to assess glucose tolerance, as described previously [13]. The rats were orally administered 2 g glucose/kg body weight following a 12 h fast. Blood samples were collected from the lateral caudal vein before administration of glucose and at 30, 60, and 120 min post-administration. The whole blood glucose concentration was measured using an amperometric quinoprotein glucose dehydrogenase electrode method (ACCU-CHEK ST meter, Roche, Tokyo, Japan). All blood samples were centrifuged at 3000 rpm for 10 min at room temperature, and the plasma was stored at − 80 °C until further use. Plasma insulin concentration was measured using an enzyme-linked immunosorbent assay kit (M1101, Morinaga, Yokohama, Japan) according to the manufacturer’s instructions. The integrated values of glucose and insulin levels, represented in terms of the areas under the curves (AUCs), were calculated using the trapezoidal method.

### Open field test

At 36 h after the oral glucose tolerance test, the open field test was conducted for assessment of anxiety-like behavior during the dark phase. All body weights were measured just before the open field test; then all rats were fasted during and after the test. The apparatus was a circular field (90 cm in diameter) surrounded by a wall (70 cm in height); it was divided into two zones, the central circle (center zone, 45 cm in diameter) and the area outside the center zone (peripheral zone). The illumination level in the center zone was 383 lx. Each rat was allowed to freely explore the field for 10 min. Upon each trial’s completion, the rat was placed back in its home cage, and the experimental field was cleaned using 70% ethanol. Each trial was recorded using an overhead digital camera (EX-ZR1000, Casio, Tokyo, Japan). The locomotive behavior and time spent in the center zone were analyzed using AnimalTracker (http://animaltracker.elte.hu [32]); rearing frequency (rising on the hind limbs) was manually analyzed.

### Tissue preparation

At 12 h after the open field test, the rats were euthanized using an overdose of sodium pentobarbital. The brains were removed, weighed, stored overnight in 4% paraformaldehyde in 0.1 M phosphate buffer, placed in 20% sucrose, and then frozen on dry ice and coronally sectioned (thickness, 30 µm) using a cryostat. Sections were stored in a cryoprotectant solution at − 30 °C until further use.

### Immunohistochemistry

For immunoperoxidase staining, free-floating sections were washed three times in phosphate-buffered saline (PBS), quenched in 2% H_2_O_2_ and 20% methanol, washed in PBS containing 0.25% Triton X-100 (PBS-T), and blocked using 3% normal horse serum for 30 min. After washing in PBS-T, the sections were incubated either with a rabbit anti-CCK antibody (1:10,000, Sigma, St. Louis, MO, USA) or a mouse anti-PV antibody (1:10,000, Sigma) in 1% blocking solution at 4 °C overnight. Thereafter, sections were washed in PBS-T and incubated with biotinylated anti-rabbit or anti-mouse antibody (1:500, Vector Laboratories, Burlingame, CA, USA) on ice for 1 h. After washing in PBS-T, the sections were reacted with avidin-biotin-peroxidase complex (ABC-Elite, Vector Laboratories), washed in PBS, and then incubated with diaminobenzidine. Finally, the sections were dehydrated in ethanol, cleared in xylene and cover-slipped with Entellan New (Merck, Darmstadt, Germany).

For immunofluorescence, the free-floating sections were processed for the antigen retrieval using citrate buffer. After blocking with 3% normal goat serum for 30 min, the sections were incubated simultaneously with rabbit anti-CCK antibody (1:4000, Sigma) and mouse anti-GAD67 antibody (1:4000, Abcam, Tokyo, Japan) at 4 °C overnight. Next, the sections were incubated with Alexa Fluor 488-conjugated goat anti-rabbit antibody (1:500, Cell Signaling Technology, Tokyo, Japan) on ice for 1 h and Alexa Fluor 555-conjugated anti-mouse antibody (1:500, Cell Signaling Technology) on ice for 2 h. The sections were mounted on MAS-coated glass slides with mounting medium containing 4′,6-diamidino-2-phenylindole (DAPI) (Vector Laboratories).

### Measurements of brain areas and cortical thicknesses

Images of whole sections were obtained using an all in one microscope (BZ-9000, Keyence, Osaka, Japan). Images of brain regions were obtained using a light microscope (BX51, Olympus, Tokyo, Japan) equipped with a digital camera (DP70, Olympus). The images were analyzed using the ImageJ software (NIH, Bethesda, MD, USA). Data were collected from both hemispheres and averaged. These measurements were conducted by an experimenter in a double-blinded fashion. To evaluate these measurements’ reliability, another experimenter as well as the experimenter who did the original experiment remeasured the brain areas and cortical thicknesses, and one-way random or two-way mixed intraclass correlation coefficients (ICCs) were calculated for intra- or inter-observer agreements.

We identified anterior to posterior (AP) 2.98 to − 4.56 mm from bregma, using Nissl staining of sections with cresyl violet with reference to the atlas [33]. First, we measured the whole area of the sections from four levels, AP 2.76, 2.28, − 0.12, and − 4.56 mm from bregma. Next, the areas of some emotion-related subregions were measured using Nissl staining of sections and comparison of CCK- or PV-stained sections. We measured the areas of the medial prefrontal cortex (mPFC), including the anterior cingulate cortex (ACC), prelimbic cortex (PL), and infralimbic cortex (IL), in two sections at AP 2.98 and 2.76 mm from bregma, and the area of the piriform cortex (Piri) in two sections at AP − 1.92 and − 2.10 mm from bregma. We measured the cortical thicknesses from two levels, the dorsolateral region of the frontal (AP 2.28) and occipital cortices (AP − 4.56 mm from bregma), according to the literature [34]. The cortical thickness was measured along the total four lines at one side: the medial elevation-line of the corpus callosum, and three additional lines 1 mm apart laterally from the medial measurement-line. These cortical thicknesses from four lines were averaged at each AP level. We measured the following subregion areas: caudate putamen (Cpu: AP 2.22 and 2.04), nucleus accumbens (Acb: AP 2.28 and 2.1 mm from bregma), amygdala [lateral amygdala (LA), basolateral amygdala (BLA), central amygdala (CeA), and medial amygdala (MeA), in two or four sections at around AP − 1.92, − 2.10, − 2.28 and − 2.46 mm from bregma], and the hippocampus [hippocampal cornu ammonis (CA) area 1 (CA1), CA2, CA3, and dentate gyrus (DG), in two sections at around AP − 2.98 and − 3.00 mm from bregma].

### Cell counting

The number of CCK and PV positive neurons were counted in the same regions for which areas were measured using the immunoperoxidase-stained sections. A neuron with detectable CCK and PV immunoreactivity above the background level was selected as a CCK  and PV positive neuron. No signals from vessels, blood cells, or reaction precipitate were counted. In the amygdala, CCK  and PV positive neurons were divided into large and small types using the following criteria: large CCK, soma diameter > 10 µm and small CCK, soma diameter ≤ 10 µm [23, 31]; large PV, rectangular diameter > 25 µm and small PV, rectangular diameter ≤ 25 µm [30]. Estimations of the neuronal density (neurons/mm^2^) were calculated from both hemispheres and averaged.

To determine whether CCK positive neurons co-express GAD67, immunofluorescent images were obtained using a confocal laser scanning microscope (FV1000, Olympus) and analyzed using a software (Olympus FluoView, Olympus). The immunofluorescent sections were used in the same regions of the measurement of the area. Only immunofluorescent positive neurons with DAPI were counted.

### Statistical analysis

Data are expressed as mean ± standard error of means. All statistical analyses were performed using SPSS statistical analysis software (IBM SPSS Statistics version 19.0, IBM Japan, Tokyo, Japan). Shapiro-Wilk and Levene’s tests were used to assess the normality and homogeneity of variance, respectively. Significant differences between groups were evaluated using Student’s *t*-test, Welch’s *t*-test, or Mann-Whitney test, and two-way ANOVA. Correlations were evaluated using Pearson’s correlation coefficient. Statistical significance was set at *p* < 0.05. Intra- or inter-observer agreements regarding both the measurements of brain areas and cortical thicknesses were assessed using the one-way random or two-way mixed ICCs. The reliability could be classified according to the ICCs: excellent (0.75 ≤ ICC); good (0.60 ≤ ICC ≤ 0.75); fair (0.40 ≤ ICC ≤ 0.60); poor (ICC ≤ 0.4) [35].

## Results

### Inclusive and pathological values

**Fig. 1:**
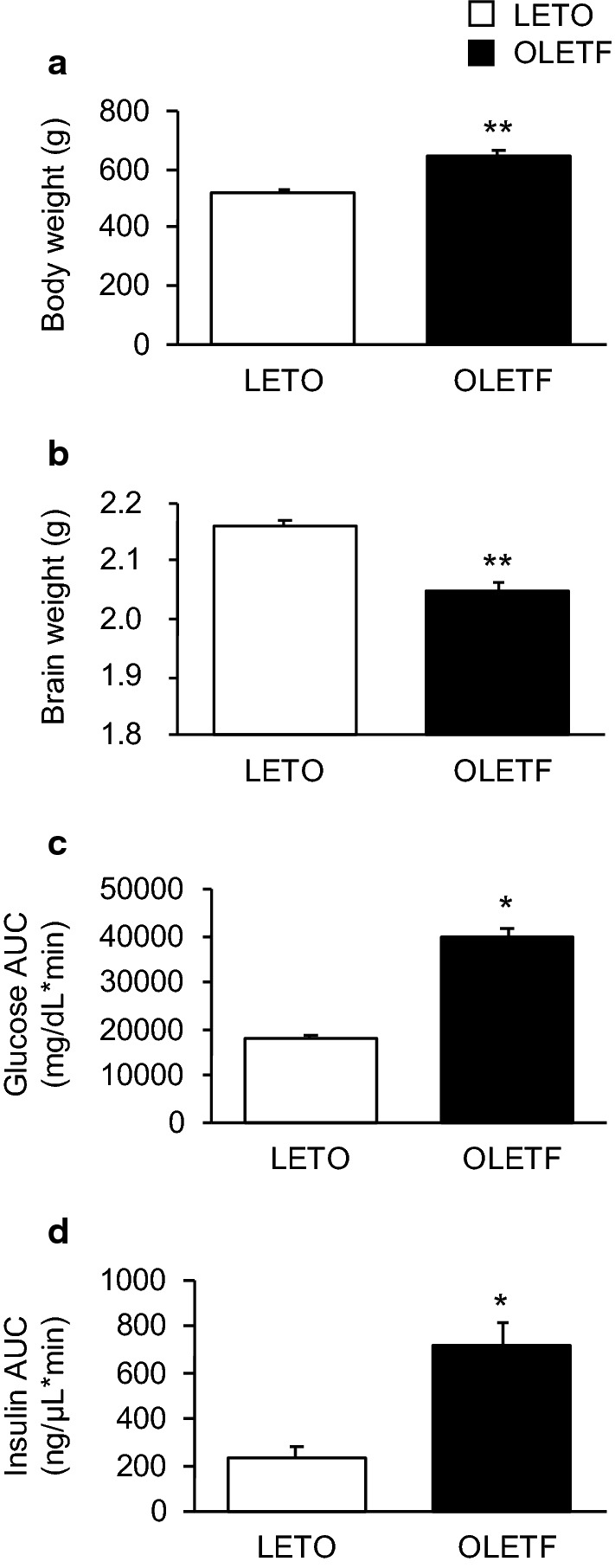
Inclusive and pathological values. Body weight (**a**) and brain weight (**b**). Areas under the curves (AUCs) of glucose (**c**) and insulin (**d**) in the oral glucose tolerance test. Values represent mean ± standard error of means. Statistically significant differences from LETO rat data are represented by asterisks: *p < 0.01 and **p < 0.001

The body weight of OLETF rats was significantly higher than that of LETO rats (*p* < 0.001; Fig. 1a); however, the brain weight of OLETF rats was significantly less than that of LETO rats (*p* < 0.001; Fig. 1b). The brain/body weight ratio percentage of OLETF rats was also significantly less than that of LETO rats (LETO: 0.45 ± 0.01%, OLETF: 0.33 ± 0.01%, *p* < 0.0001). The glucose and insulin AUCs of OLETF rats were significantly higher than those of LETO rats (*p* < 0.01 for both glucose and insulin; Fig. 1c and d).

### Measurements of brain areas and cortical thicknesses

**Fig. 2:**
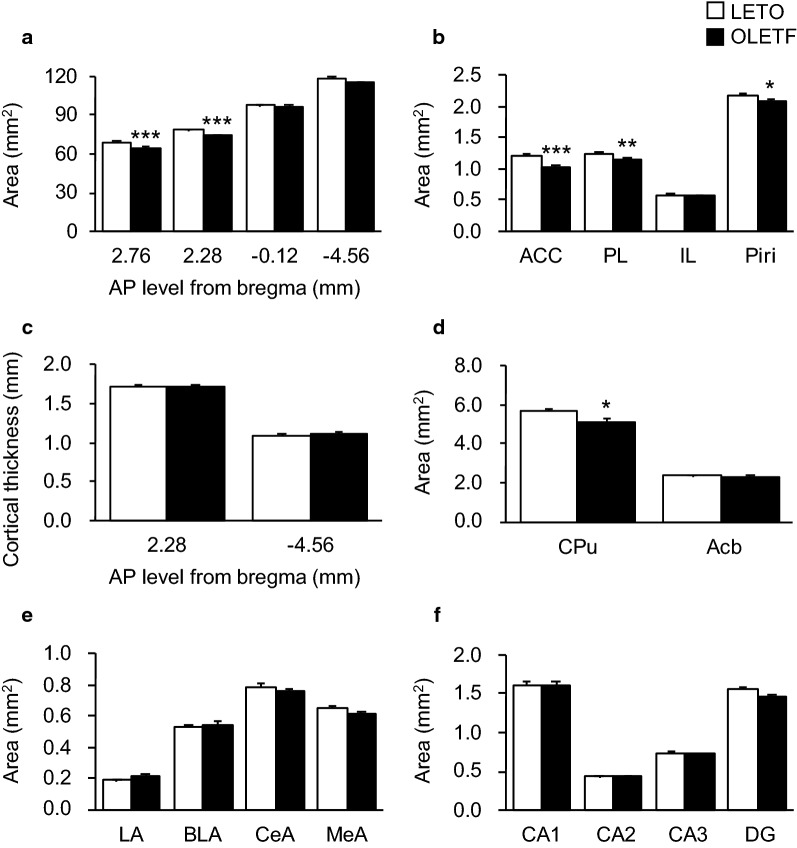
Measurements of brain areas and cortical thicknesses. Whole areas of brain sections (**a**). Areas of cortices (**b**) and dorsolateral cortical thicknesses (**c**). Areas of subregions of the forebrain (**d**), amygdala (**e**), and hippocampus (**f**). ACC: anterior cingulate cortex; PL: prelimbic cortex; IL: infralimbic cortex; Piri: piriform cortex; CPu: caudate putamen; Acb: accumbens nucleus; LA: lateral amygdala; BLA: basolateral amygdala; CeA: central amygdala; MeA: medial amygdala; CA1–3: cornu ammonis area 1–3; DG: dentate gyrus. Values represent mean ± standard error of means. Statistically significant differences from LETO rat data are represented by asterisks: *p < 0.05, **p < 0.01, and ***p < 0.001

All intra- and inter-observer ICCs were higher than 0.54 (data not shown); indicating that all measurements had excellent, good, or fine intra- and inter-observer agreement based on the classification scheme [35]. First, we quantified the whole areas of the brain sections to estimate brain-part involvement in the decreased brain weight of OLETF rats. Though the areas at AP − 0.12 and − 4.56 mm from the bregma were comparable between LETO and OLETF rats, the areas of whole sections at AP 2.76 and 2.28 mm from the bregma were significantly smaller in OLETF rats than in LETO rats (*p* < 0.001 for both AP 2.76 and 2.28 mm; Fig. 2a). Next, we quantified the brain regions’ areas and cortical thicknesses to estimate which alterations in OLETF rats. Though the area of the IL was not significantly different between LETO and OLETF rats, the areas of the ACC, PL, and Piri were significantly smaller in OLETF rats than in LETO rats (*p* < 0.001 for ACC; *p* < 0.01 for PL; *p* < 0.05 for Piri; Fig. 2b). The dorsolateral cortical thicknesses of the frontal and occipital cortex were comparable between LETO and OLETF rats (Fig. 2c). Though the area of the Acb was not significantly different between LETO and OLETF rats, the area of the CPu was significantly smaller in OLETF rats than in LETO rats (*p* < 0.05; Fig. 2d). The areas of the subregions of the amygdala and hippocampus were comparable between LETO and OLETF rats (Fig. 2e and f).

### CCK and PV positive neurons in each brain region

**Fig. 3:**
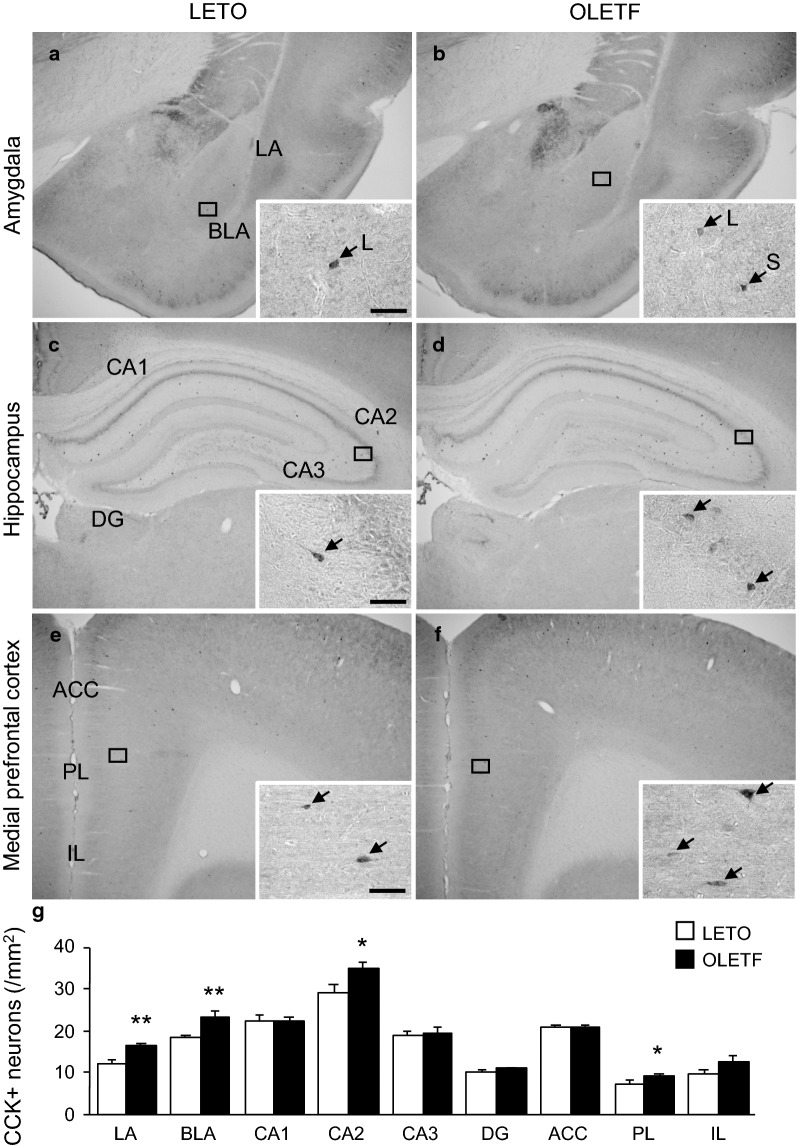
CCK positive neurons in each brain region of LETO and OLETF rats. Representative immunohistochemical images of the amygdala (**a** and **b**), hippocampus (**c** and **d**), and medial prefrontal cortex (**e** and **f**) of LETO (**a**, **c**, and **e**) and OLETF (**b**, **d**, and **f**) rats. High magnification images of the square area in each image are presented. Arrows indicate CCK positive neurons; arrows with L or S indicate large or small CCK positive neurons in the amygdala, respectively. Scale bars = 50 µm. The densities of CCK positive neurons in each region of LETO and OLETF rats (**g**). LA: lateral amygdala; BLA: basolateral amygdala; CA1–3: cornu ammonis area 1–3; DG: dentate gyrus; ACC: anterior cingulate cortex; PL: prelimbic cortex; IL: infralimbic cortex; CCK+: cholecystokinin positive. Values represent mean ± standard error of means. Statistically significant differences from LETO rat data are represented by asterisks: * *p* < 0.05, ** *p* < 0.01

**Fig. 4:**
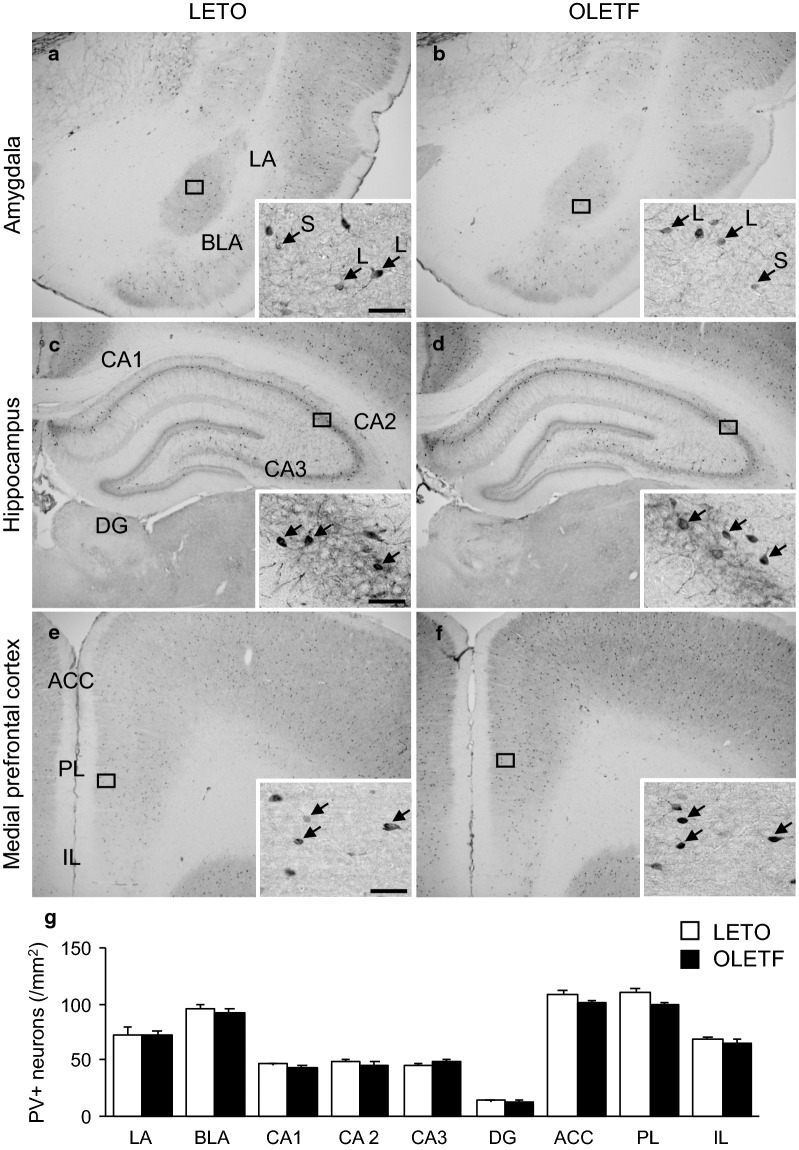
PV positive neurons in each brain region of LETO and OLETF rats. Representative immunohistochemical images of the amygdala (**a** and **b**), hippocampus (**c** and **d**), and medial prefrontal cortex (**e** and **f**) of LETO (**a**, **c**, and **e**) and OLETF (**b**, **d**, and **f**) rats. High magnification images of the square area in each image are presented. Arrows indicate PV positive neurons; arrows with L or S indicate large or small PV positive neurons in the amygdala, respectively. Scale bars = 50 µm. The densities of PV positive neurons in each region of LETO and OLETF rats (**g**). LA: lateral amygdala; BLA: basolateral amygdala; CA1–3: cornu ammonis area 1–3; DG: dentate gyrus; ACC: anterior cingulate cortex; PL: prelimbic cortex; IL: infralimbic cortex; PV+: parvalbumin positive. Values represent mean ± standard error of means

CCK and PV positive neurons were observed in the LA, BLA, hippocampus, and mPFC (Figs. 3 and 4). CCK and PV positive neurons in the LA and BLA have different characteristics according to the size of somata: large and small CCK positive neurons co-express calbindin and calretinin [23]. Rearing in an enriched environment only increases the number of small PV positive neurons, but not of large PV positive neurons [30]. Both CCK and PV positive neurons were also classified either as large or small, based on somata diameter in this study; however, because there were no differences in the alterations of large and small CCK positive and PV positive neurons between LETO and OLETF rats, we grouped the densities of large and small neurons.

OLETF rats exhibited increased densities of CCK positive neurons in the LA and BLA; there were significant main effects of animal strain (F [1, 9] = 13.3, *p* < 0.01 for LA; F [1, 9] = 13.1, *p* < 0.05 for BLA). The densities of CCK positive neurons in the LA, BLA, CA2, and PL were significantly higher in OLETF rats than in LETO rats (*p* < 0.01 for both LA and BLA, *p* < 0.05 for both CA2 and PL); conversely, no significant differences in the densities of CCK positive neurons in the CA1, CA3, DG, ACC, and IL were observed (Fig. 3g). The densities of PV positive neurons in the LA and BLA were comparable between LETO and OLETF rats: there was no significant effect of animal strain (F [1, 9] = 0.8 for LA; F [1, 9] = 0.0002 for BLA). The densities of PV positive neurons in all subregions of the amygdala, hippocampus, and mPFC were comparable between LETO and OLETF rats (Fig. 4g).

### Characterization of increased CCK positive neurons in each brain region

**Fig. 5:**
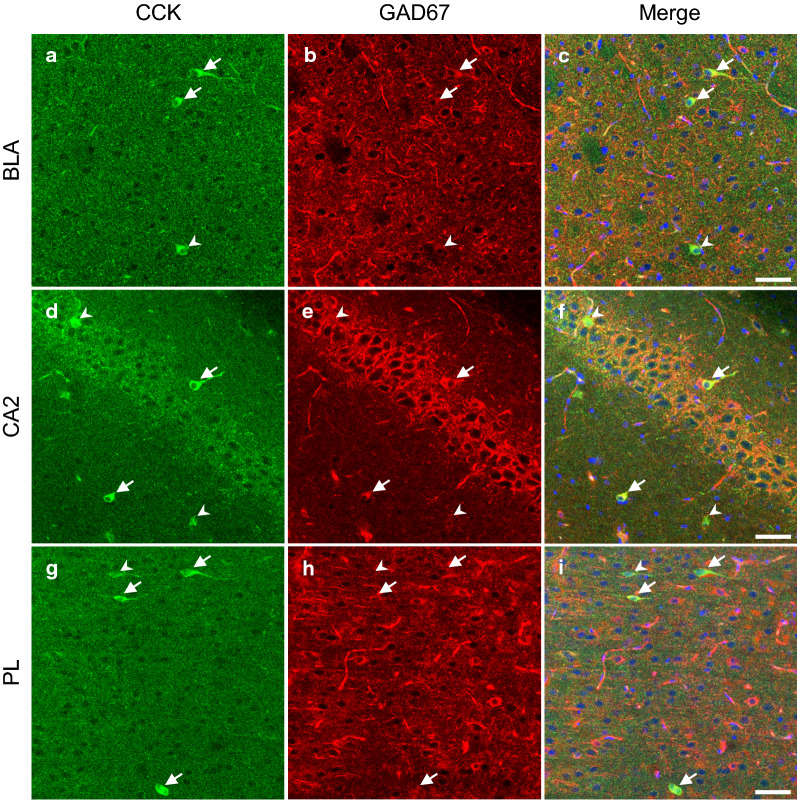
CCK and GAD67 positive neurons in each brain region of OLETF rats. Representative immunofluorescent images of the basolateral amygdala (**a**, **b**, and **c**), cornu ammonis area 2 (**d**, **e**, and **f**), and prelimbic cortex (**g**, **h**, and **i**) of OLETF rats. Labeling for CCK (**a**, **d**, and **g**) and GAD67 (**b**, **e**, and **h**) and merged images (blue for DAPI; **c**, **f**, and **i**). Arrows and arrow heads indicate CCK and GAD67 double positive neurons and CCK single positive neurons, respectively. Scale bars = 50 µm. CCK: cholecystokinin; GAD67: glutamate decarboxylase 67; BLA: basolateral amygdala; CA2: cornu ammonis area 2; PL: prelimbic cortex

We examined whether CCK positive neurons co-express GAD67 in each brain region (Fig. 5). We found that CCK positive neurons did not constantly co-express GAD67; GAD67 positive neurons accounted for 43–60% of CCK positive neurons in the amygdala, 22–72% in the hippocampus, and 64–83% in the mPFC (Table 1). The percentages of both GAD67 and CCK positive neurons in the amygdala, hippocampus, and mPFC were comparable between LETO and OLETF rats.

**Table 1:** The percentages of GAD67 positive neurons in CCK positive neurons

	LETO	OLETF
GAD67 +/CCK + neurons (%)		
LA	60.0 ± 11.3	45.6 ± 10.4
BLA	43.3 ± 16.3	46.7 ± 6.8
CA1	39.3 ± 10.3	34.7 ± 12.3
CA2	43.7 ± 6.8	32.5 ± 13.2
CA3	55.7 ± 9.8	72.2 ± 10.2
DG	34.7 ± 10.6	22.2 ± 10.0
ACC	72.8 ± 5.9	63.6 ± 5.1
PL	83.0 ± 11.8	76.4 ± 6.6
IL	73.6 ± 8.0	77.0 ± 8.3

Table shows the percentages of GAD67 and CCK positive neurons in each brain region of LETO and OLETF rats. CCK+: cholecystokinin positive; GAD67+: glutamate decarboxylase 67 positive

### Open field test

**Fig. 6:**
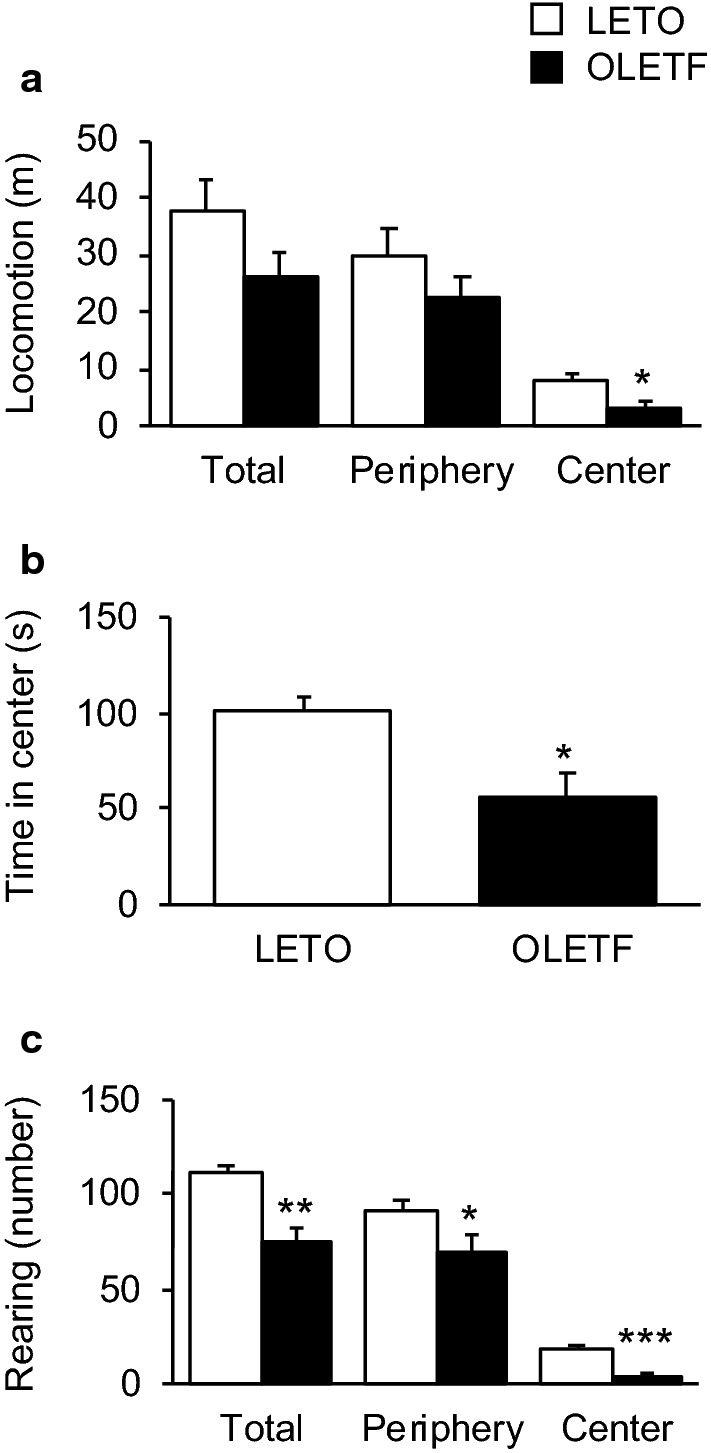
Behavioral assessment in the open field test. Locomotion in the total, peripheral, and center zones (**a**). The time spent in the center zone (**b**). The number of instances of rearing in the total, peripheral, and center zones (**c**). Values represent mean ± standard error of means. Statistically significant differences from LETO rat data are represented by asterisks: *p < 0.05, **p < 0.01, and ***p < 0.0001

Although no significant locomotion difference in the total and peripheral zones was observed, locomotion of OLETF rats in the center zone was significantly lower than that of LETO rats (*p* < 0.05; Fig. 6a). Additionally, the time spent by OLETF rats in the center zone was significantly less than that spent by LETO rats (*p* < 0.05; Fig. 6b). The ratio of locomotion to time, the mean speed, was 0.060 ± 0.008 m/s in the peripheral zone for LETO rats, 0.042 ± 0.007 m/s in the peripheral zone for OLETF rats, 0.079 ± 0.017 m/s in the center zone for LETO rats, and 0.055 ± 0.010 m/s in the center zone for OLETF rats. No significant difference in the ratio of locomotion and time was found between LETO and OLETF rats in both the periphery and center zones. The numbers of instances of rearing in the total, peripheral, and center zones were significantly less for OLETF rats (*p* < 0.01 for total; *p* < 0.05 for periphery; *p* < 0.0001 for center; Fig. 6c).

### Relationships among the parameters

First, correlation analyses were carried out to determine any association between pathological parameters of type 2 diabetes and alterations in the brain of OLETF rats. Glucose AUC significantly and negatively correlated with the whole area of brain section at AP 2.76 mm from the bregma in OLETF rats (*r* = − 0.83, *p* < 0.05), but not in LETO rats (*r* = − 0.72). There were no significant correlations between glucose AUC and other altered parameters; insulin AUC did not correlate with altered parameters as well (data not shown).

Further, we conducted correlation analyses to determine any associations related to the parameters of anxiety-like behavior. Locomotion in the center zone significantly and negatively correlated with the whole area of the brain section at AP 2.28 mm from the bregma and with the density of CCK positive neurons in the CA2 in OLETF rats (*r* = − 0.82, *p* < 0.05 for area; *r* = − 0.96, *p* < 0.01 for CCK positive neurons), but not in LETO rats (*r* = 0.08 for area; *r* = 0.10 for CCK positive neurons; Table 2). There were no significant correlations between locomotion in the center zone and other altered parameters (data not shown). Body weight significantly and negatively correlated with the number of instances of rearing in the total (*r* = − 0.90, *p* < 0.05) and peripheral (*r* = − 0.88, *p* < 0.05) zones of the open field test only in OLETF rats, but not with other altered parameters (data not shown).

**Table 2:** Relationships between an anxiety-like behavioral parameter and histological parameters

	Locomotion in center (m)	
	LETO	OLETF
Area at 2.28 mm from bregma (mm^2^)	*r* = 0.08	*r* = − 0.82 *
CCK positive neurons in CA2 (/mm^2^)	*r* = 0.10	*r* = − 0.96 **

Table shows the correlations between locomotion in the center zone in the open field test and the whole area of the brain section at AP 2.28 mm from bregma or density of CCK positive neurons in CA2 in LETO and OLETF rats. CCK: cholecystokinin. Statistical significance is represented by asterisks: *p < 0.05, and **p < 0.01

## Discussion

The main findings of this study showed that OLETF rats exhibited decreased brain weight and area, especially in the forebrain, and increased CCK levels and unchanged PV positive neuron numbers in the corticolimbic system in the early stage of type 2 diabetes; further, OLETF rats exhibited increased anxiety-like behavior in the open field test. Our data suggest an association between imbalances in the brain area and the composition of CCK positive neurons in the corticolimbic system, and anxiety-like behavior in OLETF rats.

Type 2 diabetes has been implicated in anxiety in human studies [1, 2]. Similarly, increased anxiety-like behaviors were observed in animals, such as high-sucrose diet-induced hyperglycemic rats [4] and streptozotocin-induced diabetic mice [36]. OLETF rats also reportedly exhibit increased anxiety-like behavior in the open field [17], elevated plus maze test [15–17], and hypoactivity in the open field test [17]. In the present study, OLETF rats exhibited hypoactivity, as demonstrated by decreased rearing behavior in the open field test; moreover, they exhibited increased anxiety-like behavior, as demonstrated by decreased time spent and locomotion only in the center zone in the open field test. These anxiety-induced behavioral features of OLETF rats are in agreement with the results of previous studies [15–17].

A previous study showed that the volume of the mPFC negatively correlated with the Hamilton Anxiety Rating Scales score, which is one of the assessment scales for symptoms of anxiety, in patients with obsessive-compulsive disorder [10]. Here, a significant negative correlation between the whole area of the brain section at AP 2.28 mm from the bregma and locomotion in the center zone in the open field test was observed in OLETF rats; however, the area reductions in their forebrain agree with gray matter volume reductions in type 2 diabetes and anxiety [9, 10]. Gorka et al. reported that gray matter reduction in the mPFC mediates the relationship between childhood maltreatment and trait anxiety [37]. Similarly, total gray matter volume reduction is observed in systemic inflammation-induced anxiety model rats [38]. Moreover, volume reductions in the ACC and PL were observed in chronic stress-induced anxiety model rats [39], suggesting that corticolimbic-region area reductions are involved in the anxiety-like behavioral phenotype in OLETF rats, even though the causal relationships remain unclear. Pathological processes of brain-volume reductions are heterogeneous, which include loss of neurons, glial cells, and both axons and white matter rarefaction and shrinkage [40]. Further studies are needed to evaluate what pathological processes induce brain-area reductions and the consequent functional losses associated with increased anxiety-like behavior in OLETF rats.

It is not clear what pathologies underlie the brain-area reduction in type 2 diabetes; however, inflammation and microvascular lesions were reported as the possible mechanisms [40]. Interestingly, systemic inflammation following lipopolysaccharide-induced pulmonary inflammation also induces total gray matter volume reduction [38]. In addition, high fat-diet-induced obesity without hyperglycemia and hyperinsulinemia reduces PFC volume. In the present study, there were no significant correlations between body and brain weight, and brain areas; although obesity per se might be involved in brain area reductions in OLETF rats. Glucocorticoid is generally increased in human type 2 diabetes, and corticosterone (glucocorticoid in rodents) also induces volume reductions in ACC, PL, and IL [41]. In the present study, we did not identify pathological values such as inflammatory markers, microvascular functions, and corticosterone, but our results suggest that widespread pathologies with type 2 diabetes are possibly involved in brain area reductions in OLETF rats.

CCK has been reported to be involved in anxiety-like behavior. Global chemogenetic activation of CCK positive inhibitory neurons increases anxiety-like behavior in the elevated plus maze test [27]. Similarly, experimental CCK injections in the amygdala, hippocampus, mPFC, and the cerebral ventricle increase anxiety-like behavior through the activation of the CCK-2 receptor [20, 21, 42, 43]. Moreover, CCK knock-down in the BLA reduces anxiety-like behavior in the elevated plus maze test [44]. Here, OLETF rats exhibited the increased densities of CCK positive neurons in the LA, BLA, CA2, and PL; and that in the CA2 was associated with increased anxiety-like behavior. Therefore, our results suggest that global increases of CCK positive neurons in the corticolimbic system are involved in increased anxiety-like behavior in OLETF rats, which is in agreement with the anxiogenic role of CCK in previous studies; however, the causal relationships between the increased CCK positive neurons and anxiety-like behavior remain unclear. The physiological studies using the brain slice revealed that CCK could regulate activities of several types of neurons in a cell type-specific manner via interactions with gamma amino butyric acid (GABA) and endocannabinoid systems [45, 46]. Future studies should examine the functional interactions between CCK positive neurons and other systems (i.e., GABA and endocannabinoid systems) and activities of several types of neurons each in the brain regions in which CCK positive neurons increased in OLETF rats.

OLETF rats exhibited increased CCK positive neurons in the limbic system, which appears inconsistent with the results of a previous study that the level of CCK mRNA in the cerebral cortex is comparable between LETO and OLETF rats [22]. This discrepancy may be due to the differences between these studies regarding peptide expression, which was detected using immunohistochemistry instead of mRNA expression, which in turn was detected using Northern blot analysis. Moreover, the densities of CCK positive neurons counted in the amygdala, hippocampus, and mPFC instead of the level of CCK mRNA measured only in the cerebral cortex are potentially involved in this discrepancy as well. These results suggest that CCK-expression increases particularly in the emotion-related brain regions of OLETF rats. Several studies have also reported brain region-specific alterations of inhibitory interneurons in Goto-Kakizaki rats, one of the type 2 diabetes models [26, 47]. In this study, the densities of CCK positive neurons were not associated with pathological values of type 2 diabetes in OLETF rats; however, Sherrin et al. reported that region-specific increases in CCK mRNA levels in emotion-related brain regions are induced by repeated administrations of cortagine (a corticotropin-releasing factor receptor 1-selective agonist) [48]. This study is limited to correlation analyses and a few measurements of pathological values, such as glucose and insulin, using only one stage of type 2 diabetes of OLETF rats; therefore, further studies should examine other pathological values in stages of type 2 diabetes to clarify the mechanisms of region-specific increases in CCK positive neurons in OLETF rats. Moreover, unlike Goto-Kakizaki rats, there were no alterations either in PV positive neurons or the co-expression of GAD67 in CCK positive neurons in OLETF rats. The inconsistency regarding alterations of PV and GAD67 positive neurons may be attributed to the different characteristics of type 2 diabetes between those strains: OLETF rats exhibit late onset of hyperglycemia, hyperinsulinemia, and obesity; conversely, Goto-Kakizaki rats do not exhibit these progressive properties. These results suggest that the features of type 2 diabetes would not globally affect specific neuronal populations such as CCK, PV, and GAD67 positive neurons in the early stage of type 2 diabetes in OLETF rats.

Contrary to our previous report [30], no associations were found between PV positive neurons and anxiety-like behavior in OLETF rats. A possible cause of this discrepancy may be the different experimental designs; we examined the alteration induced by an enriched environment in PV positive neurons and anxiety-like behavior in the previous report [30], whereas the alterations of type 2 diabetic OLETF rats were examined in the present study. Furthermore, the age of the rats employed by the previous report was 8 weeks old [30], whereas the age of OLETF rats used in the present study was 20 weeks old. These results suggest that alterations of PV positive neurons are inconclusive as to the underlying neuronal mechanisms of altered anxiety-like behavior.

Taken together, we have demonstrated that OLETF rats exhibit region-specific area reductions and increased CCK positive neurons in the emotion-related brain regions and increased anxiety-like behavior in the early stage of type 2 diabetes. In previous studies, patients with anxiety also exhibited brain region-specific volume reductions [10], and animal models of type 2 diabetes also exhibited cell type-specific reductions, with respect to GAD67 and PV positive neurons, in a brain region-specific manner [26, 47]. Brain region- and neuronal type-specific alterations contribute to excitation and inhibition imbalance, potentially relating to anxiety-like phenotypes in autism spectrum disorder [6]. These results indicate that imbalances of brain and neuronal composition in emotion-related regions can lead to increased anxiety-like behavior in OLETF rats. Although these findings are limited to observation at one specific time point in the present study, they still provide insight for clarifying the causal mechanisms underlying anxiety-like behavior in OLETF rats. Further studies comparing different ages are in progress to clarify the development of anxiety-like behavior in OLETF rats.

## Conclusion

In the present study, we have demonstrated that OLETF rats exhibit region-specific area reductions and increased CCK positive neurons in emotion-related brain regions and increased anxiety-like behavior in the early stage of type 2 diabetes. These results may indicate that imbalances of the brain and neuronal composition in emotion-related regions lead to increased anxiety-like behavior in OLETF rats.

## Data Availability

All data used and/or analyzed during the current study are available from the corresponding author on reasonable request.

## Abbreviations

OLETF: Otsuka Long-Evans Tokushima fatty
CCK: Cholecystokinin
PV: Parvalbumin
LETO: Long-Evans Tokushima Otsuka
GAD67: Glutamic acid decarboxylase 67
AUC: Area under the curve
PBS: Phosphate-buffered saline
PBS-T: PBS containing 0.25% Triton X-100
ICC: Intraclass correlation coefficient
DAPI: 4′,6-Diamidino-2-phenylindole
AP: Anterior to posterior
mPFC: Medial prefrontal cortex
CPu: Caudate putamen
Acb: Accumbens nucleus
Piri: Piriform cortex
ACC: Anterior cingulate cortex
PL: Prelimbic cortex
IL: Infralimbic cortex
CeA: Central amygdala
MeA: Medial amygdala
LA: Lateral amygdala
BLA: Basolateral amygdala
CA: Cornu ammonis
DG: Dentate gyrus
GABA: Gamma-aminobutyric acid
